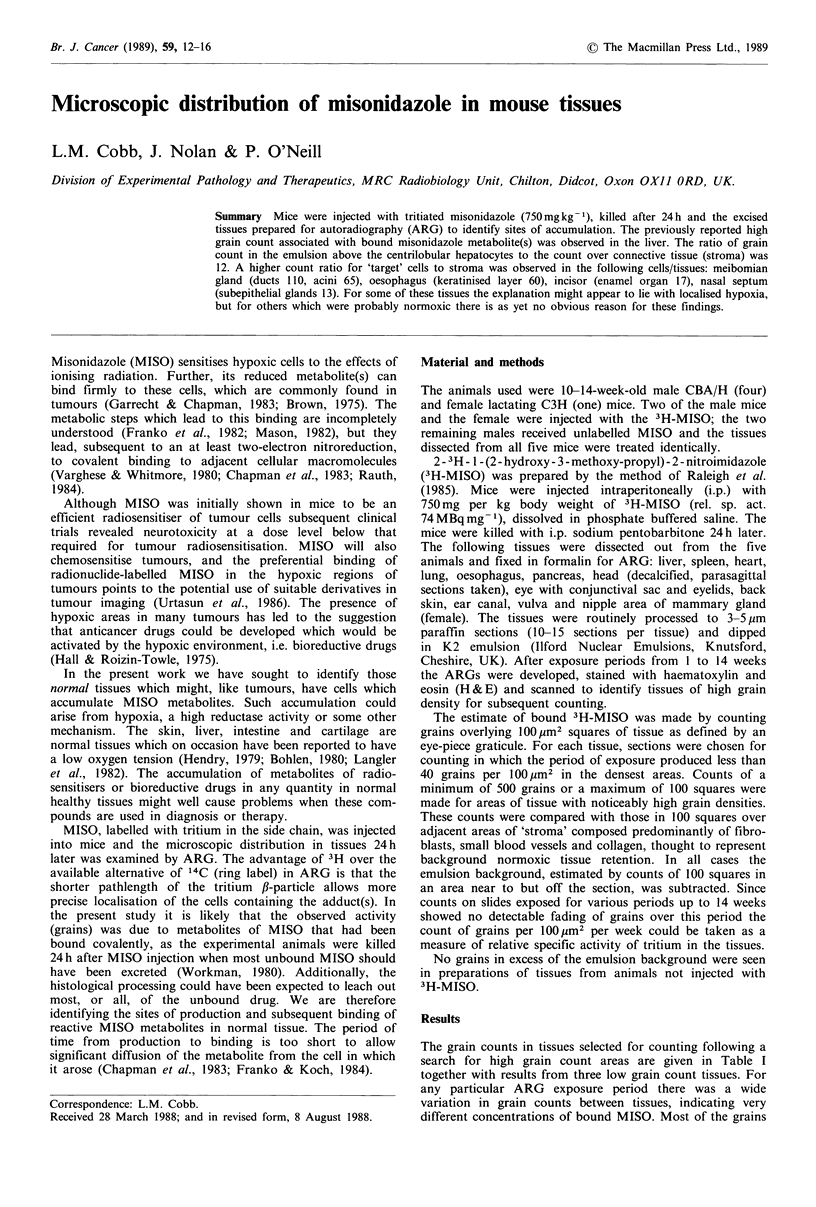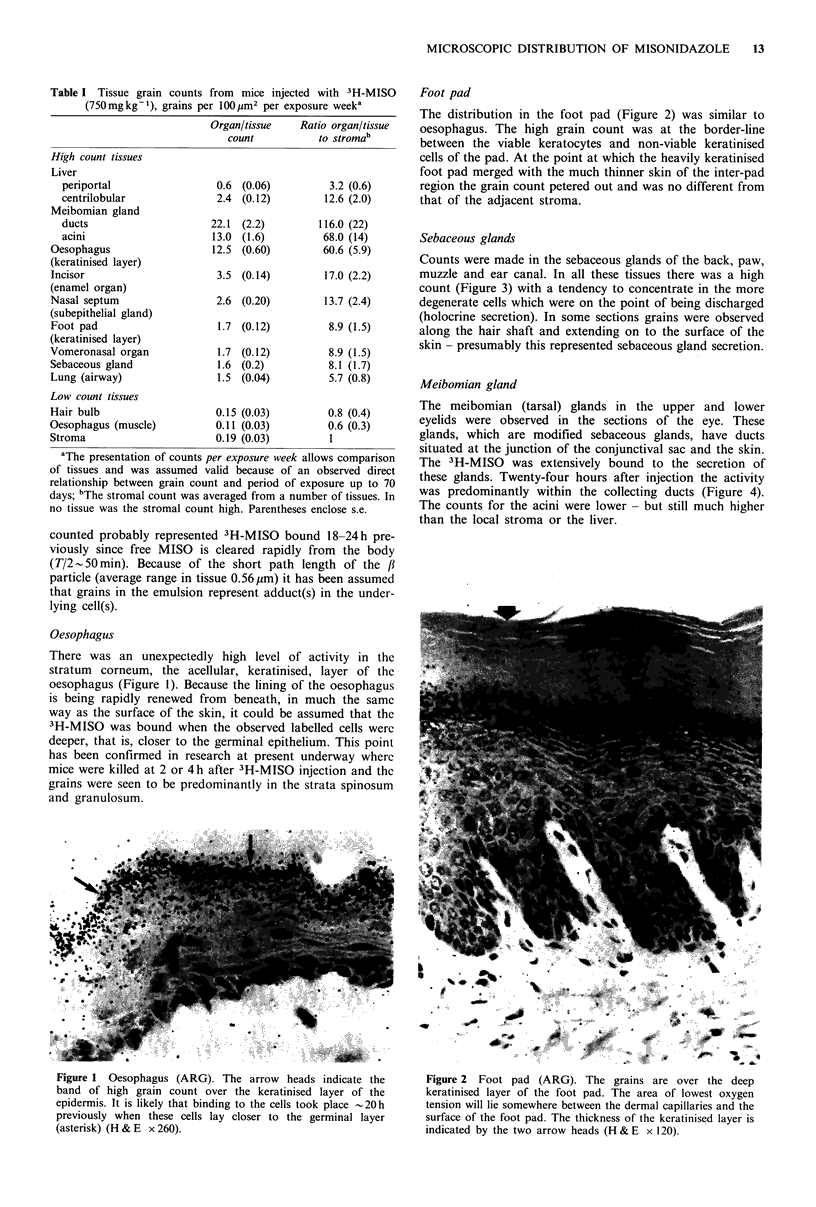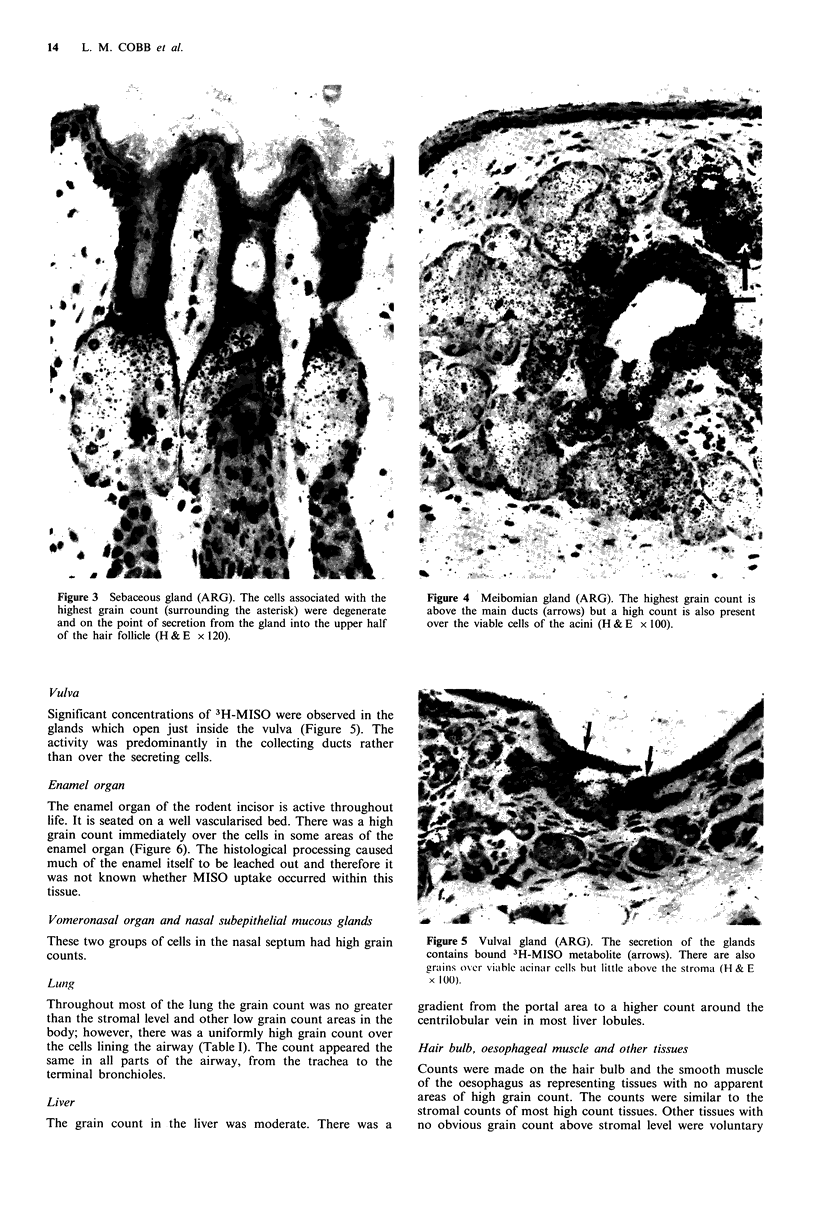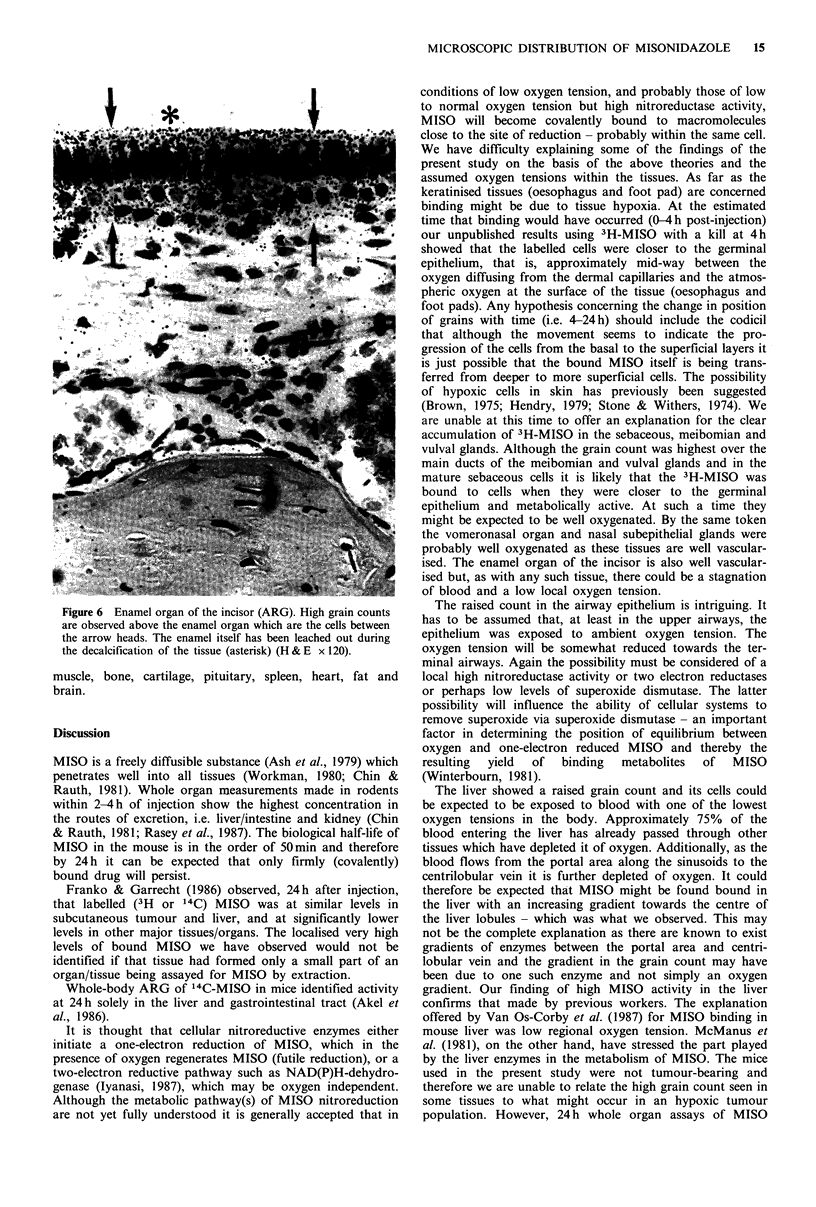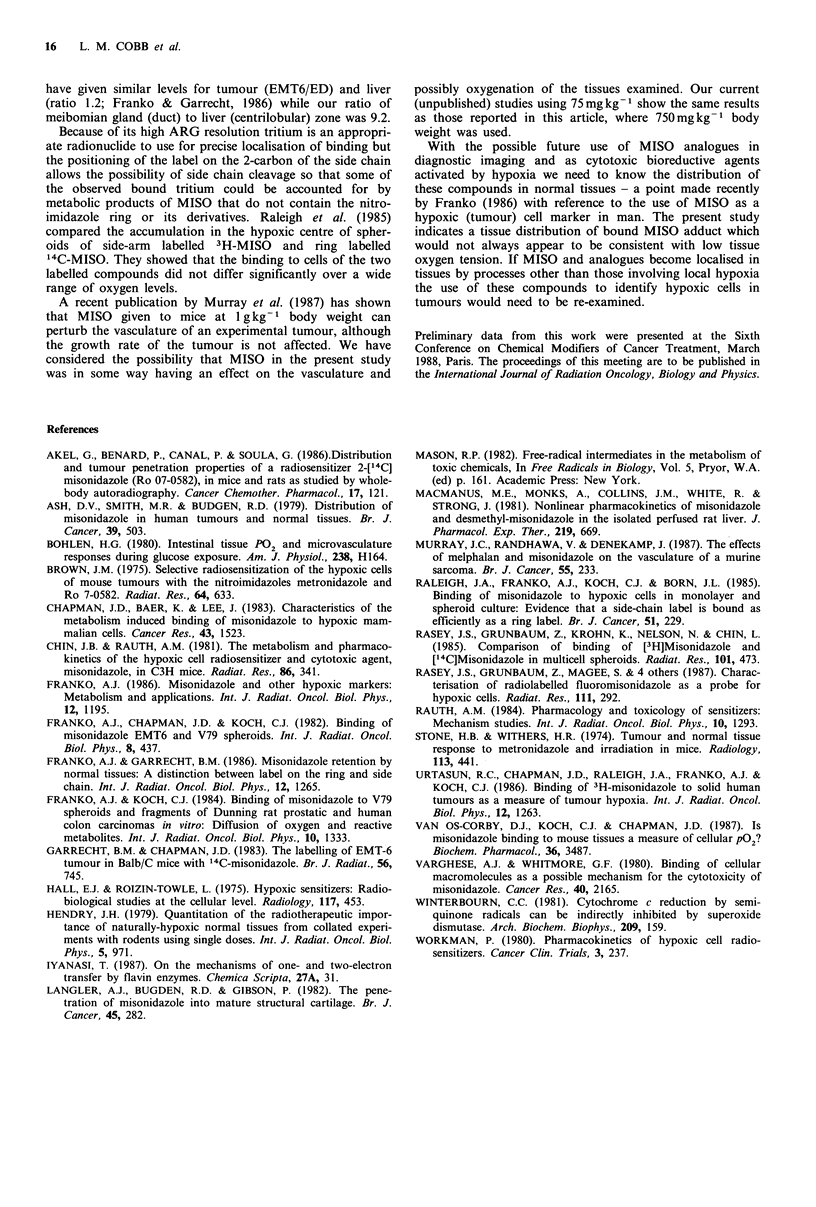# Microscopic distribution of misonidazole in mouse tissues.

**DOI:** 10.1038/bjc.1989.4

**Published:** 1989-01

**Authors:** L. M. Cobb, J. Nolan, P. O'Neill

**Affiliations:** Division of Experimental Pathology and Therapeutics, MRC Radiobiology Unit, Didcot, Oxon, UK.

## Abstract

**Images:**


Br. J. Cancer (1989), 59, 12–16 © The Macmillan Press Ltd., 1989

### Microscopic distribution of misonidazole in mouse tissues

L.M. Cobb, J. Nolan & P. O'Neill

*Division of Experimental Pathology and Therapeutics, MRC Radiobiology Unit, Chilton, Didcot, Oxon OX11 0RD, UK.*

**Summary** Mice were injected with tritiated misonidazole (750 mg kg$^{-1}$), killed after 24 h and the excised tissues prepared for autoradiography (ARG) to identify sites of accumulation. The previously reported high grain count associated with bound misonidazole metabolite(s) was observed in the liver. The ratio of grain count in the emulsion above the centrilobular hepatocytes to the count over connective tissue (stroma) was 12. A higher count ratio for 'target' cells to stroma was observed in the following cells/tissues: meibomian gland (ducts 110, acini 65), oesophagus (keratinised layer 60), incisor (enamel organ 17), nasal septum (subepithelial glands 13). For some of these tissues the explanation might appear to lie with localised hypoxia, but for others which were probably normoxic there is as yet no obvious reason for these findings.

Misonidazole (MISO) sensitises hypoxic cells to the effects of ionising radiation. Further, its reduced metabolite(s) can bind firmly to these cells, which are commonly found in tumours (Garrecht & Chapman, 1983; Brown, 1975). The metabolic steps which lead to this binding are incompletely understood (Franko *et al.*, 1982; Mason, 1982), but they lead, subsequent to an at least two-electron nitroreduction, to covalent binding to adjacent cellular macromolecules (Varghese & Whitmore, 1980; Chapman *et al.*, 1983; Rauth, 1984).

Although MISO was initially shown in mice to be an efficient radiosensitiser of tumour cells subsequent clinical trials revealed neurotoxicity at a dose level below that required for tumour radiosensitisation. MISO will also chemosensitise tumours, and the preferential binding of radionuclide-labelled MISO in the hypoxic regions of tumours points to the potential use of suitable derivatives in tumour imaging (Urtasun *et al.*, 1986). The presence of hypoxic areas in many tumours has led to the suggestion that anticancer drugs could be developed which would be activated by the hypoxic environment, i.e. bioreductive drugs (Hall & Roizin-Towle, 1975).

In the present work we have sought to identify those *normal* tissues which might, like tumours, have cells which accumulate MISO metabolites. Such accumulation could arise from hypoxia, a high reductase activity or some other mechanism. The skin, liver, intestine and cartilage are normal tissues which on occasion have been reported to have a low oxygen tension (Hendry, 1979; Bohlen, 1980; Langler *et al.*, 1982). The accumulation of metabolites of radiosensitisers or bioreductive drugs in any quantity in normal healthy tissues might well cause problems when these compounds are used in diagnosis or therapy.

MISO, labelled with tritium in the side chain, was injected into mice and the microscopic distribution in tissues 24 h later was examined by ARG. The advantage of $^3$H over the available alternative of $^{14}$C (ring label) in ARG is that the shorter pathlength of the tritium $\beta$-particle allows more precise localisation of the cells containing the adduct(s). In the present study it is likely that the observed activity (grains) was due to metabolites of MISO that had been bound covalently, as the experimental animals were killed 24 h after MISO injection when most unbound MISO should have been excreted (Workman, 1980). Additionally, the histological processing could have been expected to leach out most, or all, of the unbound drug. We are therefore identifying the sites of production and subsequent binding of reactive MISO metabolites in normal tissue. The period of time from production to binding is too short to allow significant diffusion of the metabolite from the cell in which it arose (Chapman *et al.*, 1983; Franko & Koch, 1984).

Correspondence: L.M. Cobb.
Received 28 March 1988; and in revised form, 8 August 1988.

### Material and methods

The animals used were 10–14-week-old male CBA/H (four) and female lactating C3H (one) mice. Two of the male mice and the female were injected with the $^3$H-MISO; the two remaining males received unlabelled MISO and the tissues dissected from all five mice were treated identically.

2-$^3$H-1-(2-hydroxy-3-methoxy-propyl)-2-nitroimidazole ($^3$H-MISO) was prepared by the method of Raleigh *et al.* (1985). Mice were injected intraperitoneally (i.p.) with 750 mg per kg body weight of $^3$H-MISO (rel. sp. act. 74 MBq mg$^{-1}$), dissolved in phosphate buffered saline. The mice were killed with i.p. sodium pentobarbitone 24 h later. The following tissues were dissected out from the five animals and fixed in formalin for ARG: liver, spleen, heart, lung, oesophagus, pancreas, head (decalcified, parasagittal sections taken), eye with conjunctival sac and eyelids, back skin, ear canal, vulva and nipple area of mammary gland (female). The tissues were routinely processed to 3–5 $\mu$m paraffin sections (10–15 sections per tissue) and dipped in K2 emulsion (Ilford Nuclear Emulsions, Knutsford, Cheshire, UK). After exposure periods from 1 to 14 weeks the ARGs were developed, stained with haematoxylin and eosin (H & E) and scanned to identify tissues of high grain density for subsequent counting.

The estimate of bound $^3$H-MISO was made by counting grains overlying 100 $\mu$m$^2$ squares of tissue as defined by an eye-piece graticule. For each tissue, sections were chosen for counting in which the period of exposure produced less than 40 grains per 100 $\mu$m$^2$ in the densest areas. Counts of a minimum of 500 grains or a maximum of 100 squares were made for areas of tissue with noticeably high grain densities. These counts were compared with those in 100 squares over adjacent areas of 'stroma' composed predominantly of fibroblasts, small blood vessels and collagen, thought to represent background normoxic tissue retention. In all cases the emulsion background, estimated by counts of 100 squares in an area near to but off the section, was subtracted. Since counts on slides exposed for various periods up to 14 weeks showed no detectable fading of grains over this period the count of grains per 100 $\mu$m$^2$ per week could be taken as a measure of relative specific activity of tritium in the tissues.

No grains in excess of the emulsion background were seen in preparations of tissues from animals not injected with $^3$H-MISO.

### Results

The grain counts in tissues selected for counting following a search for high grain count areas are given in Table I together with results from three low grain count tissues. For any particular ARG exposure period there was a wide variation in grain counts between tissues, indicating very different concentrations of bound MISO. Most of the grains

**Table I** Tissue grain counts from mice injected with $^{3}$H-MISO ($750\,mg\,kg^{-1}$), grains per $100\,\mu m^2$ per exposure week[a]

| | *Organ/tissue count* | *Ratio organ/tissue to stroma*[b] |
|---|---|---|
| *High count tissues* | | |
| Liver | | |
| periportal | 0.6 (0.06) | 3.2 (0.6) |
| centrilobular | 2.4 (0.12) | 12.6 (2.0) |
| Meibomian gland | | |
| ducts | 22.1 (2.2) | 116.0 (22) |
| acini | 13.0 (1.6) | 68.0 (14) |
| Oesophagus (keratinised layer) | 12.5 (0.60) | 60.6 (5.9) |
| Incisor (enamel organ) | 3.5 (0.14) | 17.0 (2.2) |
| Nasal septum (subepithelial gland) | 2.6 (0.20) | 13.7 (2.4) |
| Foot pad (keratinised layer) | 1.7 (0.12) | 8.9 (1.5) |
| Vomeronasal organ | 1.7 (0.12) | 8.9 (1.5) |
| Sebaceous gland | 1.6 (0.2) | 8.1 (1.7) |
| Lung (airway) | 1.5 (0.04) | 5.7 (0.8) |
| *Low count tissues* | | |
| Hair bulb | 0.15 (0.03) | 0.8 (0.4) |
| Oesophagus (muscle) | 0.11 (0.03) | 0.6 (0.3) |
| Stroma | 0.19 (0.03) | 1 |

[a]The presentation of counts *per exposure week* allows comparison of tissues and was assumed valid because of an observed direct relationship between grain count and period of exposure up to 70 days; [b]The stromal count was averaged from a number of tissues. In no tissue was the stromal count high. Parentheses enclose s.e.

counted probably represented $^{3}$H-MISO bound 18–24 h previously since free MISO is cleared rapidly from the body ($T/2 \sim 50$ min). Because of the short path length of the $\beta$ particle (average range in tissue $0.56\,\mu m$) it has been assumed that grains in the emulsion represent adduct(s) in the underlying cell(s).

*Oesophagus*

There was an unexpectedly high level of activity in the stratum corneum, the acellular, keratinised, layer of the oesophagus (Figure 1). Because the lining of the oesophagus is being rapidly renewed from beneath, in much the same way as the surface of the skin, it could be assumed that the $^{3}$H-MISO was bound when the observed labelled cells were deeper, that is, closer to the germinal epithelium. This point has been confirmed in research at present underway where mice were killed at 2 or 4 h after $^{3}$H-MISO injection and the grains were seen to be predominantly in the strata spinosum and granulosum.

**Figure 1** Oesophagus (ARG). The arrow heads indicate the band of high grain count over the keratinised layer of the epidermis. It is likely that binding to the cells took place ~20 h previously when these cells lay closer to the germinal layer (asterisk) (H & E ×260).

*Foot pad*

The distribution in the foot pad (Figure 2) was similar to oesophagus. The high grain count was at the border-line between the viable keratocytes and non-viable keratinised cells of the pad. At the point at which the heavily keratinised foot pad merged with the much thinner skin of the inter-pad region the grain count petered out and was no different from that of the adjacent stroma.

*Sebaceous glands*

Counts were made in the sebaceous glands of the back, paw, muzzle and ear canal. In all these tissues there was a high count (Figure 3) with a tendency to concentrate in the more degenerate cells which were on the point of being discharged (holocrine secretion). In some sections grains were observed along the hair shaft and extending on to the surface of the skin – presumably this represented sebaceous gland secretion.

*Meibomian gland*

The meibomian (tarsal) glands in the upper and lower eyelids were observed in the sections of the eye. These glands, which are modified sebaceous glands, have ducts situated at the junction of the conjunctival sac and the skin. The $^{3}$H-MISO was extensively bound to the secretion of these glands. Twenty-four hours after injection the activity was predominantly within the collecting ducts (Figure 4). The counts for the acini were lower – but still much higher than the local stroma or the liver.

**Figure 2** Foot pad (ARG). The grains are over the deep keratinised layer of the foot pad. The area of lowest oxygen tension will lie somewhere between the dermal capillaries and the surface of the foot pad. The thickness of the keratinised layer is indicated by the two arrow heads (H & E ×120).

**Figure 3** Sebaceous gland (ARG). The cells associated with the highest grain count (surrounding the asterisk) were degenerate and on the point of secretion from the gland into the upper half of the hair follicle (H & E ×120).

**Figure 4** Meibomian gland (ARG). The highest grain count is above the main ducts (arrows) but a high count is also present over the viable cells of the acini (H & E ×100).

*Vulva*

Significant concentrations of $^{3}$H-MISO were observed in the glands which open just inside the vulva (Figure 5). The activity was predominantly in the collecting ducts rather than over the secreting cells.

*Enamel organ*

The enamel organ of the rodent incisor is active throughout life. It is seated on a well vascularised bed. There was a high grain count immediately over the cells in some areas of the enamel organ (Figure 6). The histological processing caused much of the enamel itself to be leached out and therefore it was not known whether MISO uptake occurred within this tissue.

*Vomeronasal organ and nasal subepithelial mucous glands*

These two groups of cells in the nasal septum had high grain counts.

*Lung*

Throughout most of the lung the grain count was no greater than the stromal level and other low grain count areas in the body; however, there was a uniformly high grain count over the cells lining the airway (Table I). The count appeared the same in all parts of the airway, from the trachea to the terminal bronchioles.

*Liver*

The grain count in the liver was moderate. There was a gradient from the portal area to a higher count around the centrilobular vein in most liver lobules.

**Figure 5** Vulval gland (ARG). The secretion of the glands contains bound $^{3}$H-MISO metabolite (arrows). There are also grains over viable acinar cells but little above the stroma (H & E ×100).

*Hair bulb, oesophageal muscle and other tissues*

Counts were made on the hair bulb and the smooth muscle of the oesophagus as representing tissues with no apparent areas of high grain count. The counts were similar to the stromal counts of most high count tissues. Other tissues with no obvious grain count above stromal level were voluntary

**Figure 6** Enamel organ of the incisor (ARG). High grain counts are observed above the enamel organ which are the cells between the arrow heads. The enamel itself has been leached out during the decalcification of the tissue (asterisk) (H & E ×120).

muscle, bone, cartilage, pituitary, spleen, heart, fat and brain.

### Discussion

MISO is a freely diffusible substance (Ash *et al.*, 1979) which penetrates well into all tissues (Workman, 1980; Chin & Rauth, 1981). Whole organ measurements made in rodents within 2–4 h of injection show the highest concentration in the routes of excretion, i.e. liver/intestine and kidney (Chin & Rauth, 1981; Rasey *et al.*, 1987). The biological half-life of MISO in the mouse is in the order of 50 min and therefore by 24 h it can be expected that only firmly (covalently) bound drug will persist.

Franko & Garrecht (1986) observed, 24 h after injection, that labelled ($^{3}H$ or $^{14}C$) MISO was at similar levels in subcutaneous tumour and liver, and at significantly lower levels in other major tissues/organs. The localised very high levels of bound MISO we have observed would not be identified if that tissue had formed only a small part of an organ/tissue being assayed for MISO by extraction.

Whole-body ARG of $^{14}C$-MISO in mice identified activity at 24 h solely in the liver and gastrointestinal tract (Akel *et al.*, 1986).

It is thought that cellular nitroreductive enzymes either initiate a one-electron reduction of MISO, which in the presence of oxygen regenerates MISO (futile reduction), or a two-electron reductive pathway such as NAD(P)H-dehydrogenase (Iyanasi, 1987), which may be oxygen independent. Although the metabolic pathway(s) of MISO nitroreduction are not yet fully understood it is generally accepted that in conditions of low oxygen tension, and probably those of low to normal oxygen tension but high nitroreductase activity, MISO will become covalently bound to macromolecules close to the site of reduction – probably within the same cell. We have difficulty explaining some of the findings of the present study on the basis of the above theories and the assumed oxygen tensions within the tissues. As far as the keratinised tissues (oesophagus and foot pad) are concerned binding might be due to tissue hypoxia. At the estimated time that binding would have occurred (0–4 h post-injection) our unpublished results using $^{3}H$-MISO with a kill at 4 h showed that the labelled cells were closer to the germinal epithelium, that is, approximately mid-way between the oxygen diffusing from the dermal capillaries and the atmospheric oxygen at the surface of the tissue (oesophagus and foot pads). Any hypothesis concerning the change in position of grains with time (i.e. 4–24 h) should include the codicil that although the movement seems to indicate the progression of the cells from the basal to the superficial layers it is just possible that the bound MISO itself is being transferred from deeper to more superficial cells. The possibility of hypoxic cells in skin has previously been suggested (Brown, 1975; Hendry, 1979; Stone & Withers, 1974). We are unable at this time to offer an explanation for the clear accumulation of $^{3}H$-MISO in the sebaceous, meibomian and vulval glands. Although the grain count was highest over the main ducts of the meibomian and vulval glands and in the mature sebaceous cells it is likely that the $^{3}H$-MISO was bound to cells when they were closer to the germinal epithelium and metabolically active. At such a time they might be expected to be well oxygenated. By the same token the vomeronasal organ and nasal subepithelial glands were probably well oxygenated as these tissues are well vascularised. The enamel organ of the incisor is also well vascularised but, as with any such tissue, there could be a stagnation of blood and a low local oxygen tension.

The raised count in the airway epithelium is intriguing. It has to be assumed that, at least in the upper airways, the epithelium was exposed to ambient oxygen tension. The oxygen tension will be somewhat reduced towards the terminal airways. Again the possibility must be considered of a local high nitroreductase activity or two electron reductases or perhaps low levels of superoxide dismutase. The latter possibility will influence the ability of cellular systems to remove superoxide via superoxide dismutase – an important factor in determining the position of equilibrium between oxygen and one-electron reduced MISO and thereby the resulting yield of binding metabolites of MISO (Winterbourn, 1981).

The liver showed a raised grain count and its cells could be expected to be exposed to blood with one of the lowest oxygen tensions in the body. Approximately 75% of the blood entering the liver has already passed through other tissues which have depleted it of oxygen. Additionally, as the blood flows from the portal area along the sinusoids to the centrilobular vein it is further depleted of oxygen. It could therefore be expected that MISO might be found bound in the liver with an increasing gradient towards the centre of the liver lobules – which was what we observed. This may not be the complete explanation as there are known to exist gradients of enzymes between the portal area and centrilobular vein and the gradient in the grain count may have been due to one such enzyme and not simply an oxygen gradient. Our finding of high MISO activity in the liver confirms that made by previous workers. The explanation offered by Van Os-Corby *et al.* (1987) for MISO binding in mouse liver was low regional oxygen tension. McManus *et al.* (1981), on the other hand, have stressed the part played by the liver enzymes in the metabolism of MISO. The mice used in the present study were not tumour-bearing and therefore we are unable to relate the high grain count seen in some tissues to what might occur in an hypoxic tumour population. However, 24 h whole organ assays of MISO

have given similar levels for tumour (EMT6/ED) and liver (ratio 1.2; Franko & Garrecht, 1986) while our ratio of meibomian gland (duct) to liver (centrilobular) zone was 9.2.

Because of its high ARG resolution tritium is an appropriate radionuclide to use for precise localisation of binding but the positioning of the label on the 2-carbon of the side chain allows the possibility of side chain cleavage so that some of the observed bound tritium could be accounted for by metabolic products of MISO that do not contain the nitroimidazole ring or its derivatives. Raleigh *et al.* (1985) compared the accumulation in the hypoxic centre of spheroids of side-arm labelled $^{3}$H-MISO and ring labelled $^{14}$C-MISO. They showed that the binding to cells of the two labelled compounds did not differ significantly over a wide range of oxygen levels.

A recent publication by Murray *et al.* (1987) has shown that MISO given to mice at $1\,g\,kg^{-1}$ body weight can perturb the vasculature of an experimental tumour, although the growth rate of the tumour is not affected. We have considered the possibility that MISO in the present study was in some way having an effect on the vasculature and possibly oxygenation of the tissues examined. Our current (unpublished) studies using $75\,mg\,kg^{-1}$ show the same results as those reported in this article, where $750\,mg\,kg^{-1}$ body weight was used.

With the possible future use of MISO analogues in diagnostic imaging and as cytotoxic bioreductive agents activated by hypoxia we need to know the distribution of these compounds in normal tissues – a point made recently by Franko (1986) with reference to the use of MISO as a hypoxic (tumour) cell marker in man. The present study indicates a tissue distribution of bound MISO adduct which would not always appear to be consistent with low tissue oxygen tension. If MISO and analogues become localised in tissues by processes other than those involving local hypoxia the use of these compounds to identify hypoxic cells in tumours would need to be re-examined.

Preliminary data from this work were presented at the Sixth Conference on Chemical Modifiers of Cancer Treatment, March 1988, Paris. The proceedings of this meeting are to be published in the *International Journal of Radiation Oncology, Biology and Physics*.

**References**

AKEL, G., BENARD, P., CANAL, P. & SOULA, G. (1986).Distribution and tumour penetration properties of a radiosensitizer 2-[$^{14}$C] misonidazole (Ro 07-0582), in mice and rats as studied by whole-body autoradiography. *Cancer Chemother. Pharmacol.*, **17**, 121.

ASH, D.V., SMITH, M.R. & BUDGEN, R.D. (1979). Distribution of misonidazole in human tumours and normal tissues. *Br. J. Cancer*, **39**, 503.

BOHLEN, H.G. (1980). Intestinal tissue $PO_2$ and microvasculature responses during glucose exposure. *Am. J. Physiol.*, **238**, H164.

BROWN, J.M. (1975). Selective radiosensitization of the hypoxic cells of mouse tumours with the nitroimidazoles metronidazole and Ro 7-0582. *Radiat. Res.*, **64**, 633.

CHAPMAN, J.D., BAER, K. & LEE, J. (1983). Characteristics of the metabolism induced binding of misonidazole to hypoxic mammalian cells. *Cancer Res.*, **43**, 1523.

CHIN, J.B. & RAUTH, A.M. (1981). The metabolism and pharmacokinetics of the hypoxic cell radiosensitizer and cytotoxic agent, misonidazole, in C3H mice. *Radiat. Res.*, **86**, 341.

FRANKO, A.J. (1986). Misonidazole and other hypoxic markers: Metabolism and applications. *Int. J. Radiat. Oncol. Biol. Phys.*, **12**, 1195.

FRANKO, A.J., CHAPMAN, J.D. & KOCH, C.J. (1982). Binding of misonidazole EMT6 and V79 spheroids. *Int. J. Radiat. Oncol. Biol. Phys.*, **8**, 437.

FRANKO, A.J. & GARRECHT, B.M. (1986). Misonidazole retention by normal tissues: A distinction between label on the ring and side chain. *Int. J. Radiat. Oncol. Biol. Phys.*, **12**, 1265.

FRANKO, A.J. & KOCH, C.J. (1984). Binding of misonidazole to V79 spheroids and fragments of Dunning rat prostatic and human colon carcinomas *in vitro*: Diffusion of oxygen and reactive metabolites. *Int. J. Radiat. Oncol. Biol. Phys.*, **10**, 1333.

GARRECHT, B.M. & CHAPMAN, J.D. (1983). The labelling of EMT-6 tumour in Balb/C mice with $^{14}$C-misonidazole. *Br. J. Radiat.*, **56**, 745.

HALL, E.J. & ROIZIN-TOWLE, L. (1975). Hypoxic sensitizers: Radiobiological studies at the cellular level. *Radiology*, **117**, 453.

HENDRY, J.H. (1979). Quantitation of the radiotherapeutic importance of naturally-hypoxic normal tissues from collated experiments with rodents using single doses. *Int. J. Radiat. Oncol. Biol. Phys.*, **5**, 971.

IYANASI, T. (1987). On the mechanisms of one- and two-electron transfer by flavin enzymes. *Chemica Scripta*, **27A**, 31.

LANGLER, A.J., BUGDEN, R.D. & GIBSON, P. (1982). The penetration of misonidazole into mature structural cartilage. *Br. J. Cancer*, **45**, 282.

MASON, R.P. (1982). Free-radical intermediates in the metabolism of toxic chemicals, In *Free Radicals in Biology*, Vol. 5, Pryor, W.A. (ed) p. 161. Academic Press: New York.

MACMANUS, M.E., MONKS, A., COLLINS, J.M., WHITE, R. & STRONG, J. (1981). Nonlinear pharmacokinetics of misonidazole and desmethyl-misonidazole in the isolated perfused rat liver. *J. Pharmacol. Exp. Ther.*, **219**, 669.

MURRAY, J.C., RANDHAWA, V. & DENEKAMP, J. (1987). The effects of melphalan and misonidazole on the vasculature of a murine sarcoma. *Br. J. Cancer*, **55**, 233.

RALEIGH, J.A., FRANKO, A.J., KOCH, C.J. & BORN, J.L. (1985). Binding of misonidazole to hypoxic cells in monolayer and spheroid culture: Evidence that a side-chain label is bound as efficiently as a ring label. *Br. J. Cancer*, **51**, 229.

RASEY, J.S., GRUNBAUM, Z., KROHN, K., NELSON, N. & CHIN, L. (1985). Comparison of binding of [$^{3}$H]Misonidazole and [$^{14}$C]Misonidazole in multicell spheroids. *Radiat. Res.*, **101**, 473.

RASEY, J.S., GRUNBAUM, Z., MAGEE, S. & 4 others (1987). Characterisation of radiolabelled fluoromisonidazole as a probe for hypoxic cells. *Radiat. Res.*, **111**, 292.

RAUTH, A.M. (1984). Pharmacology and toxicology of sensitizers: Mechanism studies. *Int. J. Radiat. Oncol. Biol. Phys.*, **10**, 1293.

STONE, H.B. & WITHERS, H.R. (1974). Tumour and normal tissue response to metronidazole and irradiation in mice. *Radiology*, **113**, 441.

URTASUN, R.C., CHAPMAN, J.D., RALEIGH, J.A., FRANKO, A.J. & KOCH, C.J. (1986). Binding of $^{3}$H-misonidazole to solid human tumours as a measure of tumour hypoxia. *Int. J. Radiat. Oncol. Biol. Phys.*, **12**, 1263.

VAN OS-CORBY, D.J., KOCH, C.J. & CHAPMAN, J.D. (1987). Is misonidazole binding to mouse tissues a measure of cellular $pO_2$? *Biochem. Pharmacol.*, **36**, 3487.

VARGHESE, A.J. & WHITMORE, G.F. (1980). Binding of cellular macromolecules as a possible mechanism for the cytotoxicity of misonidazole. *Cancer Res.*, **40**, 2165.

WINTERBOURN, C.C. (1981). Cytochrome *c* reduction by semiquinone radicals can be indirectly inhibited by superoxide dismutase. *Arch. Biochem. Biophys.*, **209**, 159.

WORKMAN, P. (1980). Pharmacokinetics of hypoxic cell radiosensitizers. *Cancer Clin. Trials*, **3**, 237.